# Cardiomyocyte mechanodynamics under conditions of actin remodelling

**DOI:** 10.1098/rstb.2019.0081

**Published:** 2019-10-07

**Authors:** Ricardo H. Pires, Nithya Shree, Emmanuel Manu, Ewa Guzniczak, Oliver Otto

**Affiliations:** 1 Zentrum für Innovationskompetenz: Humorale Immunreaktionen bei kardiovaskulären Erkrankungen, Universität Greifswald, Fleischmannstrasse 42, 17489 Greifswald, Germany; 2 Heriot-Watt University School of Engineering and Physical Science, Institute of Biological Chemistry, Biophysics and Bioengineering, Edinburgh Campus, Edinburgh EH14 4AS, UK

**Keywords:** cardiomyocytes, contractility, cell mechanics, real-time deformability cytometry

## Abstract

The mechanical performance of cardiomyocytes (CMs) is an important indicator of their maturation state and of primary importance for the development of therapies based on cardiac stem cells. As the mechanical analysis of adherent cells at high-throughput remains challenging, we explore the applicability of real-time deformability cytometry (RT-DC) to probe cardiomyocytes in suspension. RT-DC is a microfluidic technology allowing for real-time mechanical analysis of thousands of cells with a throughput exceeding 1000 cells per second. For CMs derived from human-induced pluripotent stem cells, we determined a Young's modulus of 1.25 ± 0.08 kPa which is in close range to previous reports. Upon challenging the cytoskeleton with cytochalasin D (CytoD) to induce filamentous actin depolymerization, we distinguish three different regimes in cellular elasticity. Transitions are observed below 10 nM and above 10^3^ nM and are characterized by a decrease in Young's modulus. These regimes can be linked to cytoskeletal and sarcomeric actin contributions by CM contractility measurements at varying CytoD concentrations, where we observe a significant reduction in pulse duration only above 10^3^ nM while no change is found for compound exposure at lower concentrations. Comparing our results to mechanical cell measurements using atomic force microscopy, we demonstrate for the first time to our knowledge, the feasibility of using a microfluidic technique to measure mechanical properties of large samples of adherent cells while linking our results to the composition of the cytoskeletal network.

This article is part of a discussion meeting issue ‘Single cell ecology'.

## 1. Introduction

It is today widely accepted that changes in mechanical properties can represent an important indicator of the pathophysiological state of cells. Most technologies currently available have low throughput or can only probe small sample sizes, which limits the ability to perform interdisciplinary cell assays at the challenging interphase of biology and physics [1–3]. However, these limitations have begun to be mitigated with the arrival of a new generation of microfluidic methods such as deformability cytometry, quantitative deformability cytometry, real-time deformability cytometry (RT-DC) and real-time fluorescence and deformability cytometry that enable an analytical throughput in the range of 1000 cells per second [4–7]. Such large numbers allow for a statistical analysis based on experimental repeats with sample sizes that have previously been inaccessible with conventional methods [8,9].

RT-DC is a high-throughput technique for mechanical phenotyping of cells in suspension. Cells in a viscous phosphate buffered saline (PBS) buffer are carried across a microfluidic chip to arrive at a 300 µm long constriction with 30 µm × 30 µm cross section where they deform by hydrodynamic shear and normal stress [10]. Currently, the application of RT-DC tends to focus on the study of cells cultured in suspension and circulating cells, e.g. haematopoietic stem cells and peripheral blood cells [11,12]. While this suspended state is inherent to a number of cell types, most of the cells in the human body are part of a tissue and therefore found in an adherent state. That is the case for cardiomyocytes (CMs) which are the contracting cells present in the myocardium and the main elements responsible for cardiac activity [13].

When cultured *in vitro*, CMs can display a regular contracting behaviour that is used as a relevant indicator in toxicological studies. In fact, the mechanical and dynamic properties of CMs are tightly connected. Contractility in CMs is enabled by sarcomeres which are the supramolecular assemblies involving a combination of motor proteins such as myosin with more structural/scaffolding proteins of which actin is a notable example [14]. While sarcomeres primarily consist α-actin molecules, most cells typically express β-actin, and to a lesser extent also γ-actin, that integrate the cytoskeleton [15,16]. Cytoskeletal actin mediates processes of intracellular cargo-transport, cell adhesion and spreading, migration and represents a major contributor to the structural and mechanical stability of the cell [17]. All isoforms of actin oscillate between a monomeric globular (G) state and a polymeric filamentous (F) state that is intracellularly modulated by a plethora of regulatory and effector proteins that are responsible for the assembly and disassembly of individual filaments, but also actin filament bundling and branching [18]. Among them, formin [19] and cofilin [20] have been linked to cardiac diseases such as hypertrophic and dilated cardiomyopathies, respectively. In addition to endogenous regulators, actin dynamics can be strongly modulated by pharmacological intervention which has over the years proven to be a valuable tool to understand the cytoskeletal network [21]. One of such compounds is cytochalasin D (CytoD), a compound of fungal origin that binds to F-actin inhibiting the binding of G-actin to the F-actin barbed ends [22]. The result is the disruption of the filamentous actin network which has been extensively documented to induce cell softening in various cell types, including CMs [23,24].

Here, we explore the applicability of RT-DC to quantify the mechanical properties of CMs derived from human-induced pluripotent stem cells (hiPSC) as an adherent cell type. Advancements in stem cell research have allowed laboratories to harness the pluripotency potential of stem cells and engineer them to differentiate into specific cell types. Cardiomyocytes derived from induced pluripotent stem cells are currently an important tool in cardiac disease modelling, drug development, and in pharmacologic safety and screening studies and are of great promise for the future of personalized medicine and therapeutic interventions [25]. Indeed, hiPSC-CMs display many of the electrophysiological features that characterize primary cells [26]. However, they often lack the morphological and mechanical properties that characterize primary CMs and are typically regarded as immature cells [27].

Using RT-DC, we establish a high-throughput assay for adherent cells in suspension that enables us to determine the size and Young's modulus of hiPSC-CMs, and we demonstrate our results being in good agreement with previous reports. We then proceed to investigate the relevance of F-actin for adherent cell mechanics using CytoD as an F-actin depolymerizing agent. Our results reveal the existence of two transitions in the elastic behaviour of hiPSC-CMs characterized by a substantial decrease in Young's modulus occurring at nanomolar and micromolar concentrations of CytoD. We propose that these transitions denote alterations in cytoskeletal F-actin (at the nanomolar range), and in sarcomeric F-actin (at micromolar concentrations). In summary, we introduce a high-throughput assay for mechanical phenotyping of cardiomyocytes in suspension as an example for the label-free characterization of adherent cells of large sample size.

## 2. Material and methods

### (a) Cell culture

CMs derived from human-induced pluripotent stem cells (iCell^®^ Cardiomyocytes^2^, Fujifilm/Cellular Dynamics International) were seeded onto 24-well cell culture plates coated with 380 µl of ready-to-use Geltrex™ basement membrane extracellular matrix (Geltrex, ThermoFisher Scientific) at a density of 1 × 10^5^ cells cm^−2^ following instructions by the manufacturer. Briefly, vials of 5 × 10^6^ hiPSC-CMs were thawed and seeded using the manufacturer's cell plating medium, which was exchanged by the manufacturer's maintenance medium 4 h after seeding. Viable cells were counted with a hemocytometer and trypan blue (Biowest) as a counter stain. The cells were cultured in an incubator (37°C/5% CO_2_), the medium was exchanged every 2 days and the cells assayed on the 7th day of culture.

### (b) Sample preparation

Cells were detached via proteolytic digestion at 37°C with 190 µl of 10× concentrated TrypLE™ Select (TrypLE, ThermoFisher Scientific). After 5 min in contact with the cell monolayer, the supernatant was replaced by 190 µl of fresh enzyme solution and incubated for an additional 5 min at 37°C under orbital agitation. The reaction was stopped with the addition of 2 ml of RPMI 1640 medium without glutamine (Biowest) containing 10% fetal calf serum. The supernatant was subsequently aspirated and centrifuged at 200×*g* for 5 min, and the resulting cell pellet was re-suspended in 100 µl of MC-PBS buffer consisting of PBS (without Ca^2+^ and Mg^2+^) containing 0.6% (w/v) methyl cellulose (MC, Sigma Aldrich). The typical cell density in each sample was approximately 1 × 10^6^ cells ml^−1^.

In assays with CytoD (Sigma Aldrich), cells were exposed to different concentrations of the compound for 20 min at 37°C/5% CO_2_ before being detached. CytoD was administered to cells dissolved in maintenance medium with 2.45% (v/v) dimethyl sulfoxide (DMSO, Carl Roth GmbH). The DMSO concentration was kept constant in all treatment conditions as well as in the vehicle control. CytoD and 2.45%(v/v) DMSO were added to the MC-PBS buffer in which CMs were suspended at the concentration under investigation. The elapsed time between the commencement of sample processing until injection into the RT-DC system was set to 20 min, which was the minimal reproducible time.

Given that cell detachment from the extracellular matrix resulted in a significant amount of debris in the sample, the DNA-binding dye DRAQ5 (ThermoFischer Scientific) was employed as a means for the identification of cells. Here, 100 µl of suspended CMs were incubated at room temperature in the presence of 0.5 µl of 5 mM DRAQ5, and single intact cells were identified by fluorescence.

### (c) Real-time deformability cytometry

RT-DC was used to measure the deformation and size of suspended CMs and an analytical model was applied to derive their Young's modulus, as reported earlier [6,10]. Briefly, our RT-DC set-up (AcCellerator, Zellmechanik Dresden) is built on an inverted microscope (Axio Observer A.1, Zeiss), equipped for brightfield imaging and coupled to a laser-based fluorescence detection module (Fluorescence Module, Zellmechanik Dresden). Cell mechanical measurements are performed inside a microfluidic chip made of polydimethylsiloxane (Sylgard 184, VWR) consisting of a 300 µm long channel with a 30 µm × 30 µm squared cross section [6,28].

CMs suspended in MC-PBS buffer containing CytoD/DMSO at the concentration of interest are focused into the 300 µm long microfluidic constriction by a sheath fluid of equal composition. Cell deformation is induced by hydrodynamic shear and normal stress by applying a constant flow rate of 0.16 µl s^−1^ using a syringe pump (neMESYS, Cetoni). While normal stress arises from the pressure drop along the channel, the shear stress can be explained by the velocity gradient from maximum velocity in the centre of the channel to zero velocity at the walls [10].

Cells were imaged with a high-speed CMOS camera (MC1362, Mikrotron) operating at 2000 frames per second. Deformation *D* was calculated from brightfield images using the following equation:

$$D=1-\frac{2\sqrt{\pi A}}{P},$$

where *P* is the cell perimeter and *A* the projected cell area. Analysis was performed on-the-fly using ShapeIn software (Zellmechanik Dresden) based on measurements inside the channel and outside (reservoir) for mechanically deformed and undeformed states, respectively. Young's modulus of CMs was calculated by coupling the hydrodynamic stress distribution around the cell to linear elasticity theory and by assuming steady-state conditions [10,29].

Using the fluorescence detection capabilities of our RT-DC set-up, cells fluorescently labelled with DRAQ5 were analysed to identify cells in the presence of debris that was also detached from the extracellular matrix. Fluorescence excitation wavelength was set to 488 nm and an emission filter at 665 nm was used [7].

### (d) Cardiomyocyte imaging and contractility assay

Assessment of CM contractility was implemented using phase-contrast video microscopy. Synchronous contracting CM monolayers cultured as indicated above were imaged on an Eclipse Ti-microscope (Nikon, Japan) controlled by the NIS-Elements AR (v3.22) software (Nikon, Japan). Detection was implemented using an iXon + 897 EMCCD camera (Andor, UK) with 512 × 512 pixels. Recording was set to a period of 120 s sample^−1^ at a frame rate of 25 frames s^−1^.

### (e) Force spectroscopy

To determine the Young’s modulus of hiPSC-CMs in the adherent state, indentation experiments were performed by means of colloidal force spectroscopy using an atomic force microscope (Nanowizard 3, JPK Instruments). Tipless cantilevers (HQ:CSC38, MikroMash) with a nominal spring constant of 0.03 N m^−1^ were experimentally calibrated using the thermal method and modified by gluing a single SiO_2_ colloidal probe with 4.77 µm (±0.2 µm, s.d.) diameter (MicroParticles, GmbH) to the very tip of the lever. Cantilevers were brought into contact with cells at a speed of 2 µm s^−1^ with a force setpoint defined to 1 nN [30]. Cells seeded onto Geltrex-coated 40 mm Willco-dishes (Wilcowells) with a density of 3 × 10^5^ cells cm^−2^ were subjected to compound exposure (2.45% (v/v) DMSO and 1 µM CytoD) for 30 min at 37°C and 5% CO_2_ followed by washing the cells with the manufacturer’s maintenance medium. Measurements were subsequently carried out at room temperature in maintenance medium. Each measurement scan comprised probing 49 sites distributed as a 7 × 7 matrix over a 20 µm × 20 µm area. For each experimental condition at least 78 areas were probed across at least eight distinct measurement days (biological replicates).

### (f) Data analysis

RT-DC data processing, analysis and visualization were performed using ShapeOut software (Zellmechanik Dresden). Briefly, after defining a size range of intact CMs using fluorescence detection, an area ratio filter of 1.10 was applied to all cells to ensure a sufficient sample size. This filter compares two areas identified by the tracking algorithm: the area from the convex hull and the area from the contour. The ratio between both quantities (area ratio) is a measure of how well our software determines the real cell shape and is required to extract the CM Young's modulus [9,31].

For dose–response assays, the differential deformation was calculated which considers the initial mean cell deformation of each sample prior to the mechanical measurement and is recorded in the reservoir before the constriction. Following this procedure, an analytical model was used to calculate Young's modulus and replicates of approximately 1000 single-cell measurements were analysed using linear mixed models as published previously [9,10]. Linear mixed models assume that experimental observations can be discriminated by fixed and random effects. While random effects account for measurement bias, e.g. differences in cell concentration and culture conditions, fixed effects represent the compound-specific effect on a parameter like Young's modulus of CMs. We combined linear mixed models with permutation tests to account for the possibility of different pairings of our experimental duplicates each consisting of a control sample (vehicle control) and the treatment (compound treatment). For statistical significance, the highest *p*-value is reported.

Mean deformation data between 0 µM and 1 µM CytoD from experimental duplicates was fit to a sigmoidal equation:

$$D=A_{1}+\frac{A_{2}-A_{1}}{1+10^{(\log c_{0}-c)m_{h}}},$$

and an EC_50_ value was derived to measure the sensitivity of our assay towards cytoskeletal modifications. Here, *D* corresponds to the mean deformation of each experimental replicate at a concentration *c*, *A*_1_ and *A*_2_ represent the bottom as well as top asymptote while *c*_0_ is the EC_50_ value and *m*_h_ a measure for the hill slope of the dose–response function. Quality of the fit was determined from calculating the adjusted *R*^2^-value (OriginPro, OriginLabs).

Analysis of CM repetitive contractile motion was performed using phase-contrast video microscopy recordings to which we applied a custom-made IGOR Pro motion-detection algorithm based on the frame difference method [32]. Briefly, the algorithm finds the difference in pixel intensity between two consecutive 8-bit frames and then averages the result for an entire difference frame. The average of the difference between two consecutive frames is used as a surrogate value for the extent of motion present in the field of view. Values of duration of a contraction pulse are determined by the time taken between 5% amplitude of the contraction and relaxation peaks. Assessment of statistical significance of CM contraction dynamics was performed using Wilcoxon rank-sum test under the IGOR Pro software package.

Indentation data from colloidal force spectroscopy measurements were analysed using JPK Instruments proprietary software (v. 5.02). Force-Indentation (F-*d*) curves were fitted to the Hertz model indicated below [33]:

$$F=\frac{4ER^{1/2}}{3(1-v^{2})}\delta^{3/2},$$

that relates the variation in indentation force (*F*) at different indentation depths (*δ*) with the indented material’s Young’s modulus (*E*) given a defined Poisson’s ratio (*v*, here set to 0.5) and considers a spherical indenter with a defined radius (*R*).

Unless otherwise stated, error values and error bars reflect standard error of the mean.

## 3. Results

**Figure 1.**
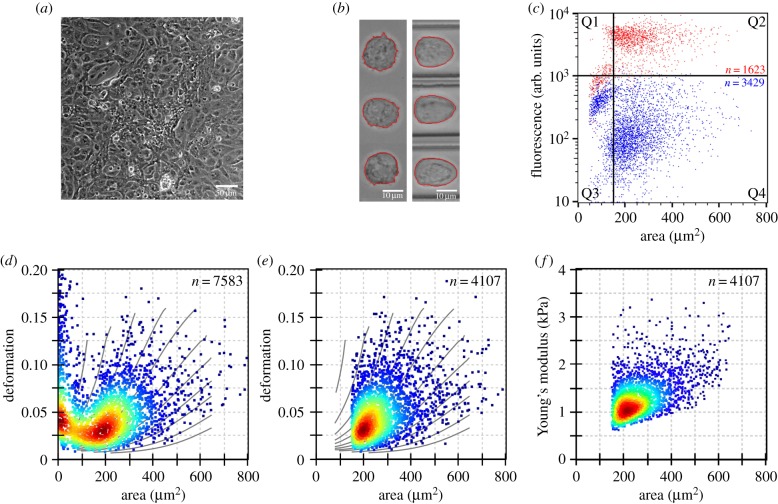
Mechanical high-throughput characterization of suspended cardiomyocytes (CMs). (*a*) Phase-contrast microscopy image of adherent human-induced pluripotent stem cell-CMs (hiPSC-CM) with a confluency above 80%. (*b*) Brightfield images of undeformed hiPSC-CM suspended in the reservoir (left) and deformed inside the constriction of the RT-DC microfluidic chip (right). Images are representative and have been taken from separate reservoir and channel measurements. (*c*) Identification of suspended CMs based on the DNA labelling dye DRAQ5. Scatter plot shows fluorescence versus cell size of unlabelled (blue) and labelled (red) cells. Debris is found at particle sizes below 150 µm^2^ or with a low fluorescence intensity (quadrants Q1/Q3), while cells are located in a size range exceeding 150 µm^2^ (quadrants Q2/Q4). (*d*) RT-DC scatter plot of CM deformation as a function of particle size. The large subpopulation of particles with a size less than 150 µm^2^ corresponds to debris. (*e*) Deformation-cell size scatter plot of intact CMs showing cells from quadrants Q2/Q4 in (*c*) after excluding small particles. (*f*) CM Young's modulus calculated from data shown in (*e*). Measurements were carried out at a flow rate of 0.16 µl s^−1^ in a channel with a 30 µm × 30 µm cross section and an area ratio filter of 1.10 was applied. (Online version in colour.)

CMs were imaged in adherent state using phase-contrast microscopy (figure 1*a*) which showed a spread-out cell shape that is typical for adherent cells. After detachment (see Material and methods), cells were re-suspended in MC-PBS buffer and analysed by RT-DC. When in suspension in the reservoir, CMs acquire a more spherical shape resulting in a smaller and circular projected area (figure 1*b*, left). After entering the central constriction, the cells deform and relax in a steady-state determined by the Poiseuille flow profile (figure 1*b*, right).

Samples obtained after detachment of CMs from the extracellular matrix comprised two populations. Aiming to differentiate intact CMs from debris particles, we resorted to fluorescence measurements integrated into our RT-DC set-up using DRAQ5 as a DNA-binding fluorophore to label cell nuclei (figure 1*c*). In an unlabelled sample (figure 1*c*, blue), two subpopulations can be clearly discerned, a subpopulation of particles with sizes up to approximately 150 µm^2^ (Q3 quadrant), and another subpopulation consisting of larger particles with sizes that range from approximately 150 µm^2^ to 800 µm^2^ (Q4 quadrant). Labelling of the sample with DRAQ5 (figure 1*c*, red) induces a very different fluorescence response in each of the two subpopulations. Only the larger particles typically show a strongly enhanced fluorescence (Q2 quadrant), which suggests a much higher DNA content compared with the smaller particles which acquire only marginal increment in fluorescence in the presence of DRAQ5 (Q1 quadrant). Therefore, larger particles (greater than 150 µm^2^) populating quadrants Q2 and Q4 are identified as cells.

In RT-DC, the mechanical properties of several thousand CMs are analysed on-the-fly. Comparison of the fluorescence (figure 1*c*) with the deformation (figure 1*d*) data from RT-DC yields a similar distribution with two subpopulations. Simultaneously, applying the size range identified above and the 1.10 area ratio filter (see Material and methods) allows the selective identification of individual hiPSC-CMs between 150 µm^2^ and 800 µm^2^. In this subpopulation, a homogeneous distribution of cell mechanical properties and cell sizes is found (figure 1*e*). A statistical analysis of two independent biological replicates using linear mixed models (see Material and methods) yields a mean deformation of 0.054 ± 0.002 and a mean projected cell area of 268.7 ± 12.7 µm^2^, corresponding to a mean diameter of 18.5 ± 2.0 µm, where each sample consists of more than 2000 single mechanical measurements. Applying the analytical model described earlier allows us to calculate the apparent Young's modulus 1.25 ± 0.08 kPa (figure 1*f*).

Having established an assay for high-throughput mechanical characterization of hiPSC-CMs in suspension, we investigated the sensitivity of real-time deformability cytometry towards cytoskeletal modifications of these cells. To this end, we used CytoD, which caps the plus end of actin filaments to induce actin depolymerization [5,34]. Because CytoD was dissolved in DMSO, we begun by evaluating the impact of the vehicle containing 2.45% (v/v) DMSO on CM elasticity. We observed a slight increase in Young's modulus to 1.30 ± 0.02 kPa in the presence of the vehicle.

**Figure 2.**
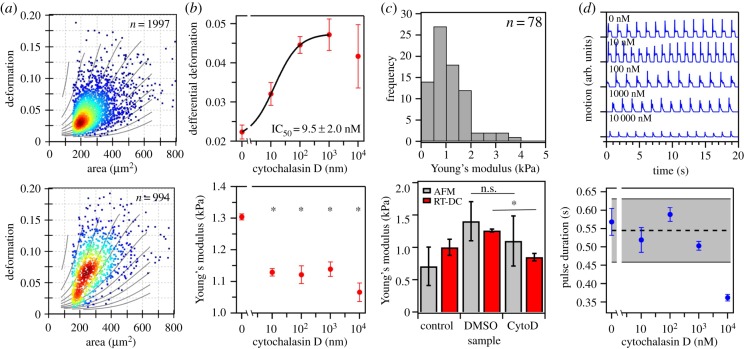
Effect of cytochalasin D (CytoD) on CM mechanics. (*a*) Deformation versus cell size scatter plot of hiPSC-CM of a 2.45% (v/v) DMSO control (top) and after treatment with 100 nM CytoD (2.45% (v/v) DMSO, bottom). (*b*) Dose–response curve of cell deformation summarizing two biological replicates for each concentration yielding a half-maximum concentration EC_50_ of 9.5 ± 2.0 nM (top). For the sigmoidal fit, data from 10^4^ nM was excluded to attribute for different regimes in mechanical properties. Bottom graph shows CM Young's modulus as a function of CytoD concentration. (*c*) AFM measurements of 78 hiPSC-CM on nine experimental replicates yield a median Young's modulus of *E* = 1.05 ± 0.35 kPa (top) and is in good agreement with RT-DC results for 2.45% (v/v) DMSO and 1 µM CytoD (bottom). (*d*) CM motion analysis using contractility assay at increasing CytoD concentrations. Each pulse describes one contraction–relaxation cycle of an entire cell monolayer (top) and contraction pulse duration of beating CMs at increasing CytoD concentrations each summarizing three biological replicates (bottom). The segmented line indicates the average pulse duration between 0 nM and 10^3^ nM CytoD, and the grey area represents the corresponding 95% confidence interval. RT-DC measurements have been carried out at a flow rate of 0.16 µl s^−1^ in a channel with a 30 µm × 30 µm cross section and an area ratio filter of 1.10 was applied. Statistical analysis was done using linear mixed models (*p < 0.05). (Online version in colour.)

We explored the impact of CytoD on hiPSC-CM mechanical properties over a concentration range covering four orders of magnitude for two experimental replicates each summarizing several thousand single-cell measurements. Adherent cardiomyocytes were exposed to CytoD for 20 min to induce actin depolymerization, followed by cell detachment and subsequent mechanical analysis. For 100 nM, RT-DC reveals a clear shift in median deformation from 0.045 ± 0.027 for the vehicle control (figure 2*a*, top) to 0.075 ± 0.037 (figure 2*a*, bottom) while cell size was not affected. Interestingly, we observe changes in cell deformation in concentrations as low as 10 nM. In these experiments, cell mechanics appears to be governed by two separate processes. While we observe a steady increase in mean differential deformation calculated from linear mixed models between 0 nM and 10^3^ nM, a lower amplitude is found for 10^4^ nM (figure 2*b*, top). A sigmoidal fit to the first range (excluding the highest concentration) yields a half-maximum concentration EC_50_ of 9.5 ± 2.0 nM (*R*^2^ = 0.94) for the effect of CytoD on the deformation of CMs (figure 2*b*, top). Calculations of the elastic properties using our analytical model and estimating the statistical significance using linear mixed models reveals the same two-step dynamics with transitions around 10 nM and 10^4^ nM, where we observe a significant decrease in Young's modulus at all concentrations when compared to the vehicle control (figure 2*d*, bottom).

In a next step, we approached the question of how well our data on CMs in suspension agrees with results using a more established method for mechanical characterization of adherent cells, such as colloidal force spectroscopy using an atomic force microscope. Performing force spectroscopy assays on eight different days and summarizing more than 78 single CM measurements we obtain a Young's modulus of *E* = 1.08 ± 0.12 kPa (figure 2*c*, top). While this confirms elastic properties obtained from RT-DC experiments, we also compared both methods for the vehicle control (2.45% (v/v) DMSO) and after treatment with 1 µM CytoD (figure 2*c*, bottom). It is noteworthy to point out that both AFM and RT-DC results follow the same trends between the different treatment conditions, however, RT-DC data shows less scattering. Remarkably, our results deviate less than 10%, which highlights the potential of RT-DC to study the mechanical properties of adherent cells in suspension.

Aiming to disentangle CM mechanics and function, we performed a beating analysis using a video microscopy approach (see Methods and methods). Within the concentration range and incubation times used in this work, an increment in CytoD concentration did not have a significant impact in the beating frequency but led to a noticeable decrease in motion signal amplitude (figure 2*d*, top). Duration of the contraction events, or pulse duration, was particularly affected but only at higher concentrations of CytoD (figure 2*d*, bottom). From 0 nM to 10^3^ nM CytoD, the duration of the contraction pulses oscillated around 0.54 ± 0.09 s (95% confidence interval highlighted by the grey band in figure 2*d*, bottom), while at 10^4^ nM CytoD we observe a decrease to 0.36 ± 0.02 s which is clearly outside the 95% confidence interval for the data collected at lower CytoD concentrations (figure 2*d*, bottom). Significance of this change was derived from concentrations between 0 nM and 10^3^ nM (*n* = 12) and compared to the data collected at 10^4^ nM CytoD (*n* = 3). Using the Wilcoxon signed-rank test, we obtained a *p*-value of 0.004, which indicates a significant difference between the two groups of data.

## 4. Discussion

The mechanical properties of cells and tissues are a key indicator of their pathophysiological state. In the heart, changes in CM elasticity are associated with dilated cardiomyopathy, hypertrophic cardiomyopathy and other forms of heart failure [35]. However, assessing the elasticity of single adherent cells commonly involves the use of low-throughput techniques where the measurement of less than 100 cells h^−1^ is common practice (e.g. [36,37]). By contrast, real-time deformability cytometry can accommodate a throughput of around 1000 cells s^−1^ but is typically limited to cells in suspension.

Here, we evaluate the use of RT-DC to probe the mechanical properties of adherent cells harvested in suspension, using cardiomyocytes derived from hiPSC-CMs as a model cell line.

Probing adherent hiPSC-CMs in RT-DC required first their detachment from the extracellular matrix. This was achieved using proteolytic digestion which led to the release not only of hiPSC-CMs, but also to a substantial amount of debris. Identification of intact CMs was achieved by DNA labelling using the DNA dye DRAQ5. This allowed us to define a range between 150 µm^2^ and 800 µm^2^ for the size of hiPSC-CMs, and therefore to exclude from our analysis all detected particles with a size below 150 µm^2^. Based on the area, we estimate an intracellular volume in the range of 2.0–17.0 pL with a mean of 3.3 ± 0.1 pL, For comparison, previous reports have estimated a volume of 3.96 ± 0.25 pL for hiPSC-CMs [38].

The mechanical analysis of primary CMs by AFM has revealed Young's moduli in the 10–15 kPa range [24,39]. Compared to primary CMs, hiPSC-CMs generally lack the high sarcomeric density found in primary cells, nor do they display a similar cytoskeletal structure [40]. Consequently, Young's modulus of hiPSC-CMs is expected to be lower than that of primary cells. In fact, according to AFM-based measurements, the average apparent Young's modulus for adherent hiPSC-CMs has been reported to be between 0.3 kPa and 0.5 kPa [41,42]. Likewise, from our RT-DC measurements we derive an average Young's modulus for individual hiPSC-CMs of 1.25 ± 0.08 kPa. The elasticity previously reported for hiPSC-CMs by AFM values resulted from the measurement of approximately 50 cells or less, whereas we measured thousands of cells in a single experimental run. Moreover, the reported AFM data compiled for each cell spreads over an entire order of magnitude. This is common in AFM measurements and is owing to the inherit sensitivity of AFM-based methods to local heterogeneities. By contrast, RT-DC applies a uniform deformation field which results in a much narrower standard deviation of Young's modulus, between 0.5 kPa and 1.5 kPa (figure 1*d*).

To further explore CM mechanics, we exposed cells to multiple concentrations of the F-actin depolymerizing drug CytoD. We observed that the vehicle alone, containing 2.45% (v/v) DMSO, increased Young's modulus of hiPSC-CMs to 1.30 ± 0.02 kPa. This cell-stiffening effect induced by DMSO has also been observed before for HL60 cells [34]. While the precise mechanism behind this effect is not entirely clear, previous work suggests that it is unlikely to stem from a stiffening of the cell membrane [43,44], but may instead involve the actin network [45–47].

The presence of CytoD substantially reduced the effect of DMSO on the mechanical properties of CMs (figure 2*a,b*). It was previously shown that CytoD leads to a decrease in the density of cytoskeletal actin filaments [48,49] and is associated with a decrease in Young's modulus after exposures of 10–30 min at concentrations in the 10^3^ nM range [50–52].

Here, we provide evidence that the mechanical susceptibility of iPSC-CMs to CytoD extends to the nanomolar range. Analysis of the RT-DC data for deformation indicates a transition in CM mechanical properties with an EC_50_ of 9.5 ± 2.0 nM while Young's modulus reveals a transition occurring at concentrations below 10 nM. The EC_50_ value for CMs is very similar to the 13.5 nM previously reported for HL60 cells using RT-DC [34]. This suggests that, despite the inexistence of sarcomeric actin in HL60 cells, part of the actin network of the two cell types shares similar properties with respect to CytoD binding. At nanomolar concentrations, it is likely that the impact of CytoD is more pronounced on dynamic actin filaments integrated in the cell cytoskeleton. In fact, in an earlier work where the actin polymerization was followed *in vitro* it was shown that CytoD can interfere with the polymerization process at concentrations in the nanomolar range within a few seconds [53].

Apart from its cytoskeleton-disrupting activity, CytoD is also known to inhibit sarcomeric contractility in cardiomyocytes. More precisely, CytoD uncouples the processes of membrane polarization [54] and calcium handling [55] from the contractile motion. The exact mechanism of inhibition of CM contraction is not entirely clear but it is envisioned that CytoD may bind to sarcomeric actin filaments thereby uncoupling cellular excitation from contractility [56]. The uncoupling activity of CytoD is typically reported at concentrations between 5 µM and 80 µM [57], which is considerably higher than values of 0.1–1 µM often used for the inhibition of cytoskeletal functions [58,59]. In part, this is owing to the fact that sarcomeric actin filaments are deemed to be more stable than the cytoskeletal ones, notably those that take part in contracting sarcomeres [14].

Indeed, our contractility assay indicates that only at high concentrations of CytoD a significantly different contractile behaviour emerges. This change is characterized by a noticeable lower amplitude of the contraction peaks (figure 2*d*, top) and a shortening in the duration of the contraction events (figure 2*d*, bottom). Changes in the duration of contraction events appear to become clearly visible only at 10^4^ nM. Between 0 nM and 10^3^ nM, the duration of contraction events appears to be evenly distributed around a mean value of 0.54 ± 0.09 s (95% confidence interval highlighted in figure 2*d*, bottom). At 10^4^ nM CytoD, the duration of contraction events is strongly reduced to a considerably lower value of 0.36 ± 0.02 s (*p* = 0.004). Interestingly, at the same concentration we also observe the onset of a third regime of mechanical stability. This third regime is characterized by an inversion in the trend of CM deformation towards smaller deformations (figure 2*c*, top) and is accompanied by a further decrease in Young's modulus (figure 2*c*, bottom). The presence of three regimes is in contrast to studies in HL60 cells where no apparent transition could be identified for concentrations of 10^4^ nM or higher. Conceivably, at these high concentrations of CytoD, damage to sarcomeric filamentous actin in cardiomyocytes will impart effects that are manifested in the level of the mechanical and dynamic properties.

Altogether, our data indicate that by using CytoD at different concentrations, we identify three regimes of mechanical stability in hiPSC-CMs. An initially unperturbed regime in the absence of CytoD, followed by a significant decrease and plateau in Young's modulus between 10 nM and 1 µM CytoD, and finally a third regime beyond 10 µM CytoD where more subtle changes in CM mechanics are observed which are accompanied by loss of contractile performance. This suggests that CytoD targets two classes of actin filaments in hiPSC-CMs. At low concentrations, CytoD primarily depolymerizes dynamic actin filaments. These are typically filaments that experience rapid turnover, are associated with the actomyosin cortex and participate in more general functions and activities. At high concentrations stable filaments as part of the sarcomere inevitably reveal a response to the presence of CytoD. If so, the amplitude of the transition at high concentrations of CytoD is anticipated to be dependent on the extent of sarcomeres present, which can represent a quantitative insight into the level of maturation of CMs.

In conclusion, using RT-DC we implemented a microfluidic assay for the mechanical analysis of adherent cells in suspension. Using hiPSC-CMs as a model cell line, we show that we are able to determine the elastic properties with high throughput and that our results are in close agreement with established methods. We were also able to describe the mechanical behaviour of CMs for different concentrations of CytoD and identify three regimes of mechanical stability between the nanomolar and micromolar range.

## Data accessibility

The datasets from this study can be found on doi:10.5061/dryad.pf012v0. The file format of the raw data is TDMS and can be read with Matlab or ShapeOut. Videos are available from the corresponding author upon reasonable request, are recorded in the ND2 format and can be read with Fiji.
